# Fabrication of SiN*_x_* Thin Film of Micro Dielectric Barrier Discharge Reactor for Maskless Nanoscale Etching

**DOI:** 10.3390/mi7120232

**Published:** 2016-12-14

**Authors:** Qiang Li, Jie Liu, Yichuan Dai, Wushu Xiang, Man Zhang, Hai Wang, Li Wen

**Affiliations:** 1 Department of Precision Machinery and Precision Instrumentation, University of Science and Technology of China, Hefei 230027, China; lqlqlq@mail.ustc.edu.cn (Q.L.); jieliu@mail.ustc.edu.cn (J.L.); daiyc@mail.ustc.edu.cn (Y.D.); xwushu@mail.ustc.edu.cn (W.X.); Zm8218@mail.ustc.edu.cn (M.Z.); 2 School of Mechanical and Automotive Engineering, Anhui Polytechnic University, Wuhu 241000, China; wanghai@ahpu.edu.cn

**Keywords:** silicon nitride, plasma enhanced chemical vapor deposition (PECVD), multilayer thin films, residual stress, micro dielectric barrier discharge, simulation

## Abstract

The prevention of glow-to-arc transition exhibited by micro dielectric barrier discharge (MDBD), as well as its long lifetime, has generated much excitement across a variety of applications. Silicon nitride (SiN*_x_*) is often used as a dielectric barrier layer in DBD due to its excellent chemical inertness and high electrical permittivity. However, during fabrication of the MDBD devices with multilayer films for maskless nano etching, the residual stress-induced deformation may bring cracks or wrinkles of the devices after depositing SiN*_x_* by plasma enhanced chemical vapor deposition (PECVD). Considering that the residual stress of SiN*_x_* can be tailored from compressive stress to tensile stress under different PECVD deposition parameters, in order to minimize the stress-induced deformation and avoid cracks or wrinkles of the MDBD device, we experimentally measured stress in each thin film of a MDBD device, then used numerical simulation to analyze and obtain the minimum deformation of multilayer films when the intrinsic stress of SiN*_x_* is −200 MPa compressive stress. The stress of SiN*_x_* can be tailored to the desired value by tuning the deposition parameters of the SiN*_x_* film, such as the silane (SiH_4_)–ammonia (NH_3_) flow ratio, radio frequency (RF) power, chamber pressure, and deposition temperature. Finally, we used the optimum PECVD process parameters to successfully fabricate a MDBD device with good quality.

## 1. Introduction

**Figure 1:**
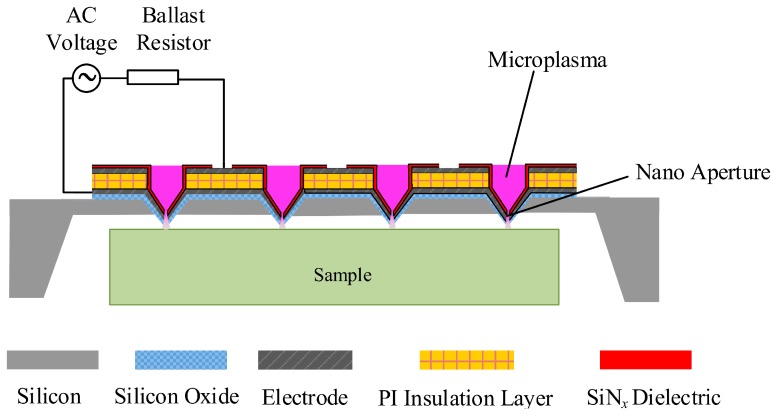
Schematic diagram of the inverted pyramid micro dielectric barrier discharge (MDBD) array for maskless nanoscale etching.

Due to its long lifetime and prevention from glow-to-arc transition, micro dielectric barrier discharge (MDBD) has wide applications, such as UV light source [1], surface modification [2,3], environmental issues [4], synthesis and etching of materials [5,6], biomedical [7], etc. Among these applications, various maskless material etching and synthesis applications have been reported [8,9,10,11,12]. As reported by Guo et al. [13], maskless etching of polymer films was realized by using an atmospheric pressure air microplasma jet. The plasma jet has the advantages of simple structure and operability under atmospheric pressure. However, at hundreds of micrometers, the etching resolution is not high, and it can only etch a single point with low efficiency. Yang et al. [14] used a paper-based microplasma array to perform maskless patterning of poly(ethylene oxide)-like thin films with a feature size down to the submillimeter scale. This microplasma device is low-cost and flexible, but the resolution is not high enough. Recently, the authors’ group proposed a novel maskless nanoscale etching method based on an inverted pyramid MDBD array. As shown in Figure 1, inverted pyramid MDBD devices are integrated into a scanning probe hollow tips array with nano-apertures at the tips. When an AC voltage is applied between the two electrodes, the microplasmas generated inside the microcavity near atmospheric pressure will eject from nano-apertures for maskless nano etching. This method has the advantages of being maskless, having high resolution, high efficiency, and low cost. The MDBD device is a multilayer structure with a structural layer, a lower electrode, an insulation layer, an upper electrode, and a dielectric layer. The selection of the dielectric barrier layer material of MDBD devices is one of the key factors, as it can prevent the electrode from being bombarded by the plasma and then sustain the high density plasmas with long lifetime. Because it possesses good dielectric, chemical resistance, and process compatibility qualities [15,16,17], SiN*_x_* is one of the most favorable dielectric barrier layer materials in MDBD devices. There are several ways to prepare SiN*_x_* films, such as direct nitridation [18], thermal evaporation [19], radio frequency (RF) sputtering [17], low pressure chemical vapor deposition (LPCVD) [16], and plasma enhanced chemical vapor deposition (PECVD) [20]. In addition to the advantage of depositing material at a relatively lower temperature, PECVD can produce a film with adjustable stress by tuning process parameters. However, the SiN*_x_* film deposited on multilayer films by regular PECVD recipe may have cracks or wrinkles when the thickness reaches a certain value, ascribed to the residual stress-induced deformation of multilayer films.

In this article, we first introduce the fabrication process of MDBD reactor and analyze the mechanism of cracks occur, then obtain the deformation of the MDBD reactor at various applied intrinsic stresses of SiN*_x_* film by 2D solid mechanics simulation. Hence, numerical simulation provides a proper intrinsic stress under which the residual stress and deformation of multilayer films is the minimum. To achieve the needed intrinsic stress, the internal relationship between some of the crucial deposition parameters, such as chamber pressure, silane (SiH_4_)–ammonia (NH_3_) flow ratio (SiH_4_/NH_3_), RF power, temperature, and the two impact response variables—namely, the level of intrinsic stress and deposition rate—is established experimentally. After tuning all variables properly, SiN*_x_* layers with high quality were successfully fabricated and applied in MDBD reactors for markless nanoscale etching.

## 2. Materials and Methods

### 2.1. Experimental Procedure

As shown in Figure 1, the inverted pyramid MDBD device has a structure of multilayer composite films. Its structural parameters and fabrication processes are similar to the microplasma device without a dielectric layer operating at DC power [21,22]. After forming inverted pyramidal microcavities by photolithography and wet etching processes, SiO_2_ layers were grown on both sides of the wafer by thermal oxidation. Next, two metal Ni electrode and polyimide (PI) insulation layers were deposited and patterned step by step. Then, the SiN*_x_* dielectric barrier layer was deposited by PECVD system (System100, Oxford Instruments, Yatton, UK and PD-220, SAMCO, Kyoto, Japan). Finally, the wafer was back-released, and the nano apertures on the tips were fabricated by Focused Ion Beam (Helios NanoLab650, FEI, Hillsboro, OR, USA). Note that the equipment of Oxford Instruments (plasma excitation frequency 13.56 MHz) could only produce a SiN*_x_* film with tensile stress, while the facility of SAMCO (plasma excitation frequency 308 kHz) yielded a compressive stress state of the deposited layer. The reason for this phenomenon is the difference in the plasma excitation frequency of the two pieces of equipment. The plasma excitation frequency influences the material intrinsic stress by influencing the ion bombardment energy. The lower the excitation frequency, the more the ions in the plasma can oscillate according to the alternating electric field, and thus transfer energy to the grown silicon nitride film, resulting in densification. In this case, the film is compressively stressed compared to the substrate. At high frequencies, not all of the ions can follow the alternating field, so the membrane is not dense and exhibits tensile stress [23].

The experiment investigating the relationship between the intrinsic stress, deposition rate, and deposition parameters consists of three parts: wafer cleaning, film deposition, and film characterization. The reactant gas used in the film deposition experiment was argon diluted to 10% silane and pure ammonia, and the nitrogen gas flow was fixed at 400 sccm all the time. X-ray photoelectron spectroscopy (XPS) (ESCALAB 250, Thermo Fisher Scientific, Waltham, MA, USA) was used to determine the Si/N ratio of SiN*_x_* films. The residual stresses in the multilayer thin films plate due to the SiO_2_ structural layer, Ni electrode layers, PI Insulation layer, and SiN*_x_* dielectric layer were computed by monitoring the change in the bending radius of unpatterned wafers through mechanical profilometer (Dektak XT, Bruker, Billerica, MA, USA). The residual stress results for each film are shown in Table 1. It should be noted that the stress of the Si substrate layer is negligible [24]. The deposition rate was calculated by first measuring the thickness of the deposited SiN*_x_* layer using a reflective spectral thickness meter (SRM300-M200, Angstrom Sun Technologies, Boston, MA, USA) divided by the deposition time. The morphology and microstructure of the dielectric layer were studied using field emission scanning electron microscopy (SIRION200, FEI, Hillsboro, OR, USA).

### 2.2. Numerical Model

**Figure 2:**
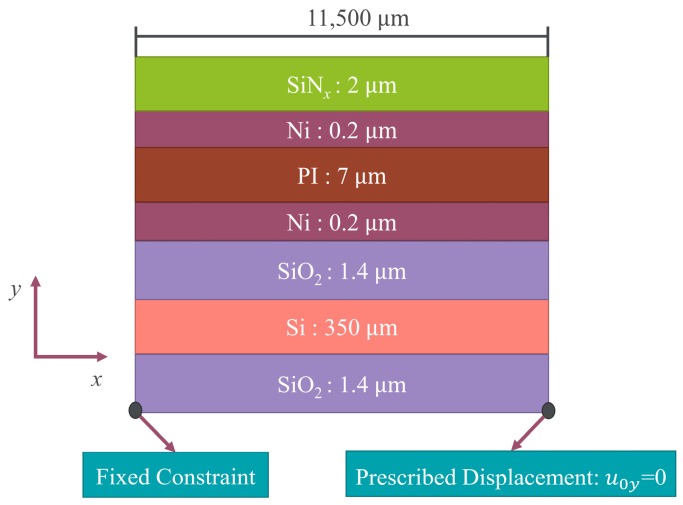
Two-dimensional plane stress approximation geometric model for MDBD device.

A 2D geometry of the multilayer thin films plate (Figure 2) based on COMSOL 5.1 (Comsol Multiphysics GmbH, Gottingen, Germany) was used with the plane stress approximation for numeric efficiency (no stress in the direction of thickness). Here the bottom-left corner of the multilayer plate was fixed, and the bottom-right corner of the plate was constrained in the *y* direction, which prevents rigid-body movements but did not affect the stress distribution. The analysis used two steps: first, the Si substrate layer, SiO_2_ layer, Ni layer, and PI layer were active. The SiN*_x_* film was not active in this step. In step two, all seven layers were active, and the temperature was dropped from 300 °C to room temperature (20 °C). The residual stresses (Table 1) of each layer were used as initial stresses in the simulation process. Then, the residual stress of device could obtain after coupling the thermal stresses by setting the temperature change in the thermal expansion interface. Other basic material properties, such as density, Poisson’s ratio, Young’s modulus, and coefficient of thermal expansion (CTE) are also shown in Table 1. The layer thicknesses and the size of the plate are shown in Figure 2.

## 3. Results and Discussion

### 3.1. Simulation Results

**Figure 3:**
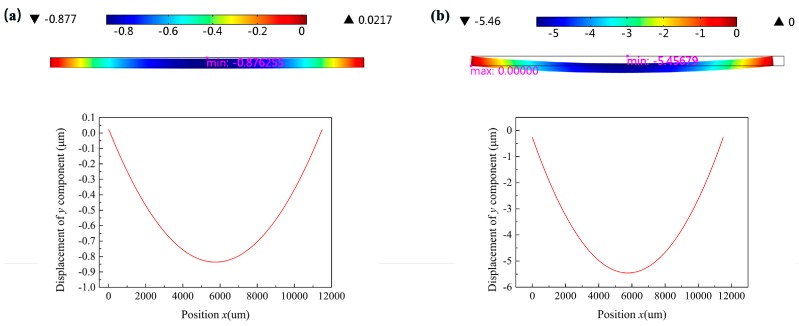
Displacement profiles of *y* component of the multilayer thin films. (**a**) Before deposition of SiN*_x_*; (**b**) After deposition of SiN*_x_* film by plasma enhanced chemical vapor deposition (PECVD) with the original recipe (Plasma System100, Oxford Instruments).

The residual stress consists of thermal stress and intrinsic stress. Thermal stress arises from the mismatch of the coefficient of thermal expansion (CTE) between the thin films and the substrate, and the intrinsic stress is generally associated with processes occurring during film growth. The CTEs of SiO_2_, Si, SiN*_x_*, PI, and Ni are 5 × 10^−7^, 2.6 × 10^−6^, 2.3 × 10^−6^, 3.5 × 10^−5^, 1.3 × 10^−5^, respectively [25]. When a silicon nitride film is deposited at a high temperature of about 300 °C, the device may cause severe stress and deformation due to severe mismatches in the CTE of several materials. Figure 3a demonstrates the initial displacement profile of the device before the deposition of SiN*_x_*. We can see that the maximum deformation of the device is less than a micron. However, the maximum deformation of the device soared to 5.5 μm after a 2-μm-thick SiN*_x_* layer was deposited by PECVD (Plasma System100, Oxford Instruments) with 500 MPa intrinsic tensile stress, as shown in Figure 3b. It can be seen from the theoretical and numerical simulation analysis that the deformation of the device increases greatly after the deposition of SiN*_x_*, which may cause cracks in the device.

**Figure 4:**
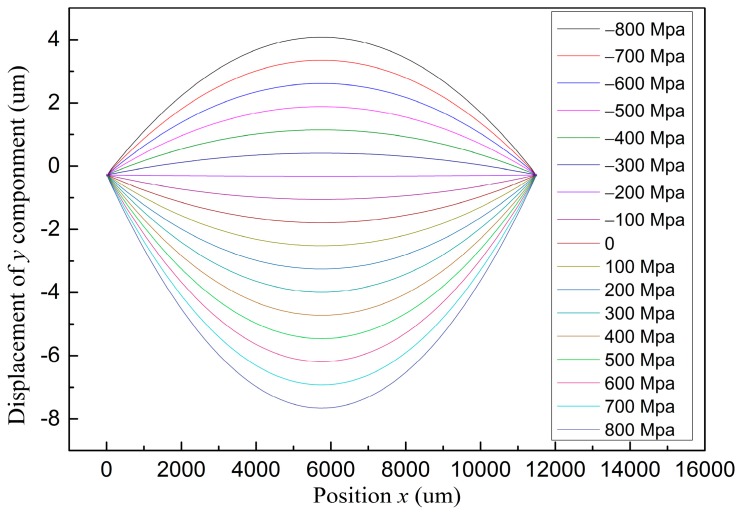
Displacement profiles of *y* component of the multilayer thin films under intrinsic stresses of SiN*_x_* film ranging from −800 MPa compressive stress to 800 MPa tensile stress, with stress increment of 100 MPa.

In order to balance the residual stress of multilayer thin films and minimize the deformation of the microplasma device, we investigated the deformation profiles of thickness (*y*) direction with various intrinsic stresses of SiN*_x_* film ranging from −800 MPa compressive stress to 800 MPa tensile stress, with stress increment of 100 MPa. As shown in Figure 4, the multilayer plate profile changed from up-convex curve to concave-down curve gradually, while the stress of SiN*_x_* increased from −800 MPa to 800 MPa. It is worth noting that the deformation of the multilayer films has a minimum of 0.3 μm when the intrinsic stress of SiN*_x_* is about −200 MPa. Thus, the simulation result points the way to the optimization of a SiN*_x_* film deposition process.

### 3.2. Influence of the Key Process Parameters on the Intrinsic Stress Level and Deposition Rate

Different PECVD equipment and deposition parameters may bring different SiN*_x_* stress [24]. In order to obtain −200 MPa intrinsic compressive stress of SiN*_x_* to get minimum deformation of the multilayer structure device, we selected the PECVD facility of SAMCO, which can yield a compressive stress film of SiN*_x_*. However, the magnitude of stress will be changed with the adjustment of process parameters. So, we aimed to establish the fundamental relationships between the level of intrinsic stress as well as deposition rate and some of the crucial deposition parameters, such as chamber pressure, SiH_4_/NH_3_ ratio, RF power, temperature, and the two impact response variables, by means of experimental methods. Note that the residual stress of SiN*_x_* deposited in unpatterned wafer is nearly intrinsic stress because of the virtually equal CTEs of Si and SiN*_x_* (2.6 × 10^−6^ vs. 2.3 × 10^−6^).

**Figure 5:**
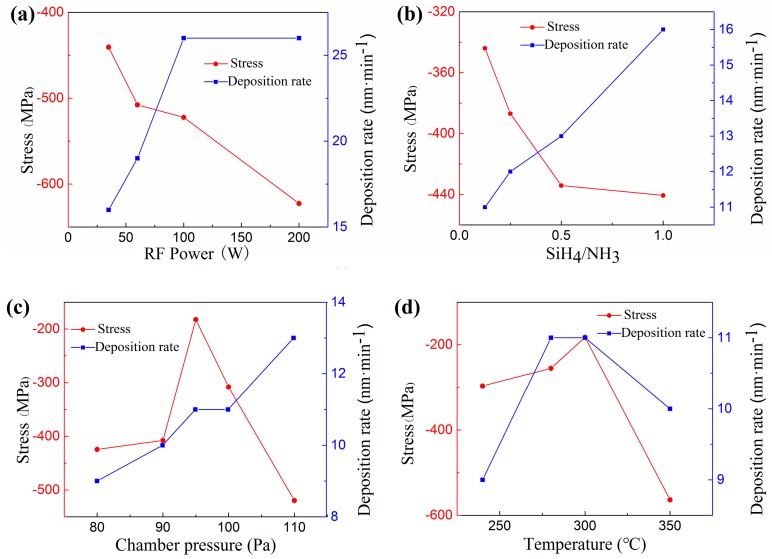
Influence of some of the key deposition parameters of the process: (**a**) RF power; (**b**) SiH_4_–NH_3_ flow ratio; (**c**) Chamber pressure; (**d**) Temperature, with the intrinsic stress level and deposition rate. The equipment used here was PECVD (PD-220, SAMCO).

The established relationships are shown in Figure 5. The influence of the RF power on the level of intrinsic stress and deposition rate was investigated, with the SiH_4_/NH_3_ ratio, pressure, and temperature kept constant at 100/10, 100 Pa, and 300 °C, respectively. As shown in Table 2 and Figure 5a, both the intrinsic stress within the SiN*_x_* layer and the deposition rate increase with RF power. A reasonable explanation for these trends is that with an increase in RF power, the average energy of the electrons per unit volume in the vacuum chamber increases [26], so that the number of reactive species in the plasma increases, causing the deposition rate to increase. An increase in the energy of reactive species in the plasma will result in the ion bombardment effect [27]. With higher power, the ion bombardment effect is more serious, so the film is in the compressive stress state and the stress increases as the power increases.

The same as the above rule, both the intrinsic stress within the SiN*_x_* layer and the deposition rate increase with the increase in SiH_4_–NH_3_ flow ratio, as shown in Table 3 and Figure 5b. Power was kept at 35 W in this section of the experiment, and pressure and temperature were kept at 100 Pa, 300 °C, respectively. The reason for the increase in intrinsic stress is that there is a higher Si–N atomic ratio with the increase in SiH_4_–NH_3_ flow ratio, and this results in surplus of silicon atoms in the film, then the film Si/N ratio far from the standard stoichiometric ratio, 0.75. The deposition rate increases, since there is a higher proportion of silicon-related species within the plasma, and hence a higher amount of reactants for deposition [28].

Table 4 and Figure 5c depicts the established relationship with chamber pressure under the experimental conditions: power: 35 W, SiH_4_/NH_3_: 100/80, temperature: 300 °C. As can be observed, the intrinsic stress first decreases and then increases, and the deposition rate increases with pressure. This is because when the reaction pressure is low, the frequency of electron collision per unit volume in the vacuum chamber is low, so the electrons are mainly in a high energy state. The number of high energy electrons across the activation and ionization thresholds increases. Thus, the energy carried by the active species in the plasma is increased and causes a severe ion bombardment effect. Therefore, the stress of the SiN*_x_* film is large under low reaction pressure. As the reaction pressure continues to increase, the electron collision frequency will be increased, the particle carrying energy will be reduced, the ion bombardment effect will be weakened, and the film stress will become lower. When the pressure inside the vacuum chamber is too high, the collision frequency of electrons and particles in the unit volume will become larger. The effect of the deposition rate increasing and the frequent collision energy exchange between the substrate and the particles cannot be neglected. The film stress will again increase.

Finally, the influence of temperature is presented in Table 5 and Figure 5d. The stress decreases while deposition rate increases in the first stage, and then the stress increases and deposition rate decreases, on the contrary. At low deposition temperatures, the stress of the silicon nitride film is high due to the atoms in the film having insufficient energy to diffuse to the most suitable position, and the many defects caused by the large amount of hydrogen (H) in the film. However, as the temperature increases, the interstices or tiny voids in the film are sintered and shrink, and the H content in the film decreases and the Si–N bond increases, so that the film stress decreases. As the deposition temperature continues to increase, as the grain growth in the film, it will make the film stress re-increase. When the deposition temperature is changed from 300 °C to 350 °C, the deposition rate decreases, with the substrate temperature increasing. This is because when the temperature of the substrate is raised, the ability of atoms adsorbed by the surface becomes lower. The density of the silicon nitride film is increased due to the film shrinkage caused by the decrease of the H content. Therefore, the film deposition rate decreases slightly with the increase of deposition temperature, but the overall effect is relatively weak.

**Figure 6:**
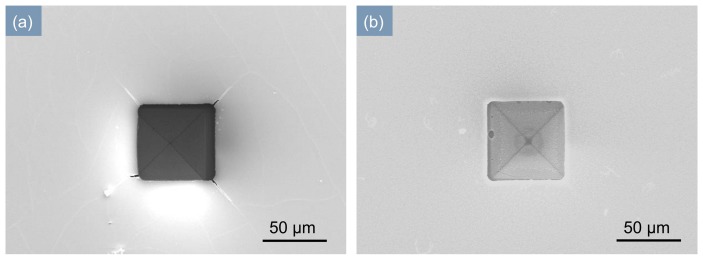
SEM images of inverted pyramid MDBD device. (**a**) After deposition of 2 μm-thick-SiN*_x_* by original recipe of PECVD (Plasma System100); (**b**) After deposition of 2 μm-thick-SiN*_x_* by optimized recipe of PECVD (PD-220, SAMCO).

Considering the stress and deposition rate of SiN*_x_* film, after fine tuning all variables, the combination of parameters applied finally is: SiH_4_/NH_3_/N_2_: 100/80/400, chamber pressure: 95 Pa, RF power: 35 W, and temperature: 300 °C. The measured stress value is −182.4 MPa, and the deposition rate is 11 nm/min. We then applied these optimized deposition parameters to fabricate the SiN*_x_* dielectric barrier layer of a MDBD device. Figure 6 is the scanning electron microscopy (SEM) images before and after the deposition of SiN*_x_*. We can see that the cracks are apparent in the film prepared with the original recipe, with about 500 MPa intrinsic tensile stress (Figure 6a), and the surface morphology of the device with the optimized recipe is excellent (Figure 6b).

## 4. Conclusions

The crack forming mechanism of the SiN*_x_* dielectric barrier layer of a micro dielectric barrier discharge reactor was analyzed by numerical simulation. The desired intrinsic stress of the SiN*_x_* layer was obtained by 2D solid mechanics simulation, and the required film was successfully prepared by tuning the key PECVD deposition parameters, thus compensating the intrinsic bending of the multilayer structure microplasma reactor. In the future, the 2-μm-thick SiN*_x_* will be used as a layer protecting the metal electrode from being bombarded by the plasma to prolong reactor life-span in maskless nanoscale fabrication.

## Figures and Tables

**Table 1:** The basic material properties in the simulation process. CTE: Coefficient of thermal expansion; PI: Polyimide.

Film	Si	SiO_2_	Ni	PI	SiN*_x_*
Density (kg/m^3^)	2329	2200	8900	1300	3100
Poisson’s ratio	0.28	0.17	0.31	0.42	0.23
Young’s modulus (GPa)	170	70	219	3.1	250
CTE (1/K)	2.6 × 10^−6^	5 × 10^−7^	1.3 × 10^−5^	3.5 × 10^−5^	2.3 × 10^−6^
Residual stress (MPa)	0	−480	200	30	(−800, 100, 800)

**Table 2:** Influence of RF power with the intrinsic stress level and deposition rate.

RF Power (W)	Deposition Rate (nm·min^−1^)	Intrinsic Stress (MPa)
35	16	−440.6
60	19	−507.8
100	26	−522.3
200	26	−622.5

Note: SiH_4_/NH_3_/N_2_: 100/10/400, pressure: 100 Pa, temperature: 300 °C.

**Table 3:** Influence of SiH_4_/NH_3_ with the intrinsic stress level and deposition rate.

SiH_4_/NH_3_	Deposition Rate (nm·min^−1^)	Si/N	Intrinsic Stress (MPa)
100/80	11	0.70	−344.0
100/40	12	0.83	−386.9
100/20	13	0.94	−434.1
100/10	16	1.11	−440.6

Note: Power: 35 W, pressure: 100 Pa, temperature: 300 °C, N_2_: 400 sccm.

**Table 4:** Influence of chamber pressure on the intrinsic stress level and deposition rate.

Chamber Pressure (Pa)	Deposition Rate (nm·min^−1^)	Intrinsic Stress (MPa)
80	9	−424.4
90	10	−407.5
95	11	−182.4
100	11	−308.0
110	13	−519.6

Note: power: 35 W, SiH_4_/NH_3_/N_2_: 100/80/400, temperature: 300 °C.

**Table 5:** Influence of temperature on the intrinsic stress level and deposition rate.

Temperature (°C)	Deposition Rate (nm·min^−1^)	Intrinsic Stress (MPa)
240	9	−296.9
280	11	−255.3
300	11	−182.4
350	10	−563.7

Note: Power: 35 W, SiH_4_/NH_3_/N_2_: 100/80/400, pressure: 95 Pa.
